# Bacteriology

**Published:** 1905-04-01

**Authors:** 


### Progress in Medicine and Surgery.

### BACTERIOLOGY.

**Leprosy.**—Rost[1] has succeeded in growing acid-fast bacteria, and amongst these the leprosy bacillus, by using medium freed from chlorides. The medium was a broth obtained by distilling beef-extract, but has lately been manufactured by passing superheated steam over beef-extract soaked in pumicestone in bottles inside the autoclave. In such a medium the bacillus tuberculosis grows in from one to three days, and the leprosy bacillus in from three to five days, the latter forming a curdy, white, stringy, heavy deposit at the bottom of the tubes. A solid medium was also obtained free from chlorides by dialysing nutrient agar in frequently-changed warm distilled agar. The following facts concerning the leprosy bacillus have been demonstrated. It retains the stains of acid dyes longer than any other bacillus of its class; it contains small oval spores which give it a beaded appearance; it is capable of growing as long branching filaments; it is found inside epithelial cells. The tubes were generally inoculated from the fresh granulations of ulcers. A toxin (leprolin) was prepared by filtering cultures through Pasteur candles, reducing the fluid to one-tenth the original bulk and again filtering. When injected into leprous subjects it gives rise to a marked reaction, which is accompanied by a return of sensation in the anæsthetic patches, by relief of the shooting pains and rapid cicatrisation of the ulcers. Leprolin for curative purposes should be injected once a fortnight in doses of 10 ccs. Rost states that he has examined many samples of badly-cured fish for leprosy bacilli, but always unsuccessfully, and from the large amount of salt in such fish, he thinks that it is highly improbable that they could form a nidus for the germ.

**Immunity.**—Welsh[2] in an address on modern theories of immunity, devotes his attention chiefly to Ehrlich's theory. According to Ehrlich's view the interaction of toxin and antitoxin is of a chemical nature, the two substances combining to form a harmless inert product. That the action is chemical and not biological is supported by Martin's work on snake venom, and by the fact that the chemical body ricin can be shown to form an antibody, and the disintegrating effect of the ricin and the protective action of the antiricin can be demonstrated in test-tubes containing susceptible blood-corpuscles. Ehrlich next showed that the complete toxin molecule contained two chemical affinities, one (haptophore) can combine with a corresponding affinity in the antitoxin molecule, while the other (toxophore) can exert a poisonous action on the tissues. Some toxins have an especial preference for certain tissues such as the nervous system. The haptophore atom groups not only have an affinity for antitoxins but also for receptors (side-chains) of the cell protoplasm which are normally concerned with fixing molecules of food-stuff. This affinity for certain tissue substances is evidenced by the fact that a mixture of tetanus toxin and guinea-pig's brain is harmless, whilst the mixture of any other organ of the guinea-pig with tetanus toxin is as toxic as ever. Under the stimulus of repeated doses of toxin there is a persistent, progressive, and finally excessive regeneration of receptors which become thrown off into the blood and body fluids, and meet and neutralise fresh atoms of toxin. In some diseases, such as enteric fever and pneumonia, the invading bacilli are widely distributed through the system, and to resist these the body has need not only of antitoxins, but also of substances that agglutinate the bacteria and other substances that disintegrate and destroy them (bacteriolysins). The bacteriolysins are made up of two bodies, one (the intermediary) is relatively stable and not destroyed by heat, the other, lacking in both of these characteristics, is known as the "complement." The intermediary links the destructive complement to the susceptible bacterium, and then bacteriolysis follows. Cells other than bacteria can set up this reaction, for instance blood-cells, nerve-cells, etc., may form specific hæmolysins, neuro-lysins, leuco-lysins and nephrolysins, the dissolution of the cells being accomplished by appropriate complements linked to them by appropriate intermediaries. Neither agglutins nor precipitins are strictly specific in their action; for example, a concentrated typhoid agglutinin may agglutinate cultures of bacilli other than the bacillus typhosus. Both complement and intermediary are developed from leucocytes, and Metchnikoff traces the origin of Ehrlich anti-bodies to phagocytic cells. The varying views with regard to the phenomena of immunity were well illustrated in the discussion at the Oxford meeting of the British Medical Association.[3] Muir maintained that for every given quantity of immune body fixed by blood cells only a given quantity of complement can be taken up, and that both immune body and complement can be taken up by the particles of blood cells disintegrated by hæmolysis. Madsen, in direct opposition to Ehrlich's teaching, demonstrated that the toxin-antitoxin reaction is reversible, and that many very powerful toxins have a comparative low affinity for antitoxin. Dreyer, from the standpoint of physical chemistry, showed that the phenomena which had been interpreted as showing the existence of agglutinoids really were due to an error in not allowing a sufficient time reaction. Valuable contributions to the discussion were also made by Wright, Noguchi, Robert Muir, and C. J. Martin. Wassermann[4] states that every toxic substance for which a specific serum can be obtained has the property of firmly fixing itself to the body cell, becoming a portion of its protoplasm from which it cannot be abstracted by chemical solvents. Tetanus poison is such a toxic body and in this way differs from such bodies as strychnine, which only enters into loose combination with the cells of the central nervous system. This fixation of the toxic substance is due to its special receptor group of atoms, whilst its toxophore group excites a peculiar reaction in the cell and the cell gives rise to a group of proliferated receptors which become free in the blood plasma and which are the true 'anti-bodies. These anti-bodies when employed in immunisation have the effect of combining with toxins and rendering them inert. Tetanus toxin travels to the central nervous system along the nerves; tetanus antitoxin travels in the blood stream. Some micro-organisms have more than one kind of molecule carrying the same toxin capable of being assimilated by different receptors in the cells of the patient. For such organisms the antisera should be prepared from several cultures injected into a variety of animals. He has found that the necessity for a polyvalent serum applies in the case of typhoid fever. He suggests, too, that in diphtheria if a mixed serum were dried, put up in tablets, and given to patients to suck during convalescence it would destroy the bacilli which may be present in the throat and nasopharynx.

[1] Brit. Med. Jour., Feb. 11, 1905. [2] Scot. Med. and Surg. Jour., October and November, 1904. [3] Brit. Med. Jour., Sept. 10, 1904. [4] Lancet, Nov. 19, 1904, p. 1440.

